# Sibling competition, dispersal and fitness outcomes in humans

**DOI:** 10.1038/s41598-023-33700-3

**Published:** 2023-05-09

**Authors:** Aïda Nitsch, Charlotte Faurie, Virpi Lummaa

**Affiliations:** 1 Department of Biology, University of Turku, Turku, Finland; 2 Institute for Advanced Study in Toulouse, Université Toulouse 1 Capitole 1, Esplanade de l’Université, 31080 Cedex 6, Toulouse, France; 3 Department of Animal and Plant Sciences, University of Sheffield, Sheffield, S10 2TN UK; 4 University of Montpellier, Montpellier, France; 5 Institute of Evolutionary Sciences, Centre National de la Recherche Scientifique, Place Eugène Bataillon, CC 065, 34095 Montpellier Cedex 5, France

**Keywords:** Anthropology, Zoology, Behavioural ecology, Evolutionary ecology

## Abstract

Determining how sibling interactions alter the fitness outcomes of dispersal is pivotal for the understanding of family living, but such studies are currently scarce. Using a large demographic dataset on pre-industrial humans from Finland, we studied dispersal consequences on different indicators of lifetime reproductive success according to sex-specific birth rank (a strong determinant of dispersal in our population). Contrary to the predictions of the leading hypotheses, we found no support for differential fitness benefits of dispersal for either males or females undergoing low vs. high sibling competition. Our results are inconsistent with both hypotheses that family members could have different fitness maximizing strategies depending on birth rank, and that dispersal could be mainly driven by indirect fitness benefits for philopatric family members. Our study stresses the need for studying the relative outcomes of dispersal at the family level in order to understand the evolution of family living and dispersal behaviour.

## Introduction

Natal dispersal (the departure from the natal site) is an important life-history trait influencing family living, demography, and population genetic structure^1,2^. Consequently, understanding both the causes and consequences of natal dispersal is central to a number of diverse disciplines, including ecology, sociology, genetics or demography^3^, as well as for governments concerned with predicting and managing human migration patterns or evaluating the impact of migration policies. Among factors associated with dispersal patterns (probability, distance and timing of dispersal), kin have a critical effect^4–6^. Indeed kin competition and/or cooperation influence the relative costs and benefits of dispersal and philopatry (the settlement at the natal site)^7^. Moreover, inclusive fitness theory predicts that dispersal strategies could be beneficial both directly via, for example, an increased access to mating opportunities, and indirectly via, for instance, a decrease in kin competition for limited resources between philopatric kin^6,8^. Characteristics of philopatric and dispersing individuals vary at the intra-familial level^7,9,10^, which suggests that family members might differ in their fitness maximizing strategies^11^. For instance, parental favouritism, competitive asymmetries between siblings^12,13^ or personality differences^14,15^ influence an individual’s access to fitness benefits for some family members whereas for others, philopatry might be more beneficial.

However, theoretical predictions on the outcomes of dispersal depending on kin interactions are broad. To evolve, fitness of dispersers should generally be the same as that of residents, but this is not necessarily predicted in the case of kin competition^7^. For instance when including kin presence, some models predict that dispersal can evolve without any benefits to the dispersing kin when the benefits for the philopatric kin outweigh the dispersal costs for the dispersing individuals^4,16^. On the contrary, other models suggest that philopatry could be promoted when kin interactions are linked to fitness benefits, as in social species^6,17^. However, these models do not include detailed information of intra-familial dynamics or of asymmetries between siblings, and thereby prevent making clear predictions on the outcomes of dispersal behaviour, intra-familial dynamics, fitness outcomes and other life-history traits. Similarly, although empirical studies comparing fitness outcomes of dispersing and philopatric individuals exist^18–21^, the potential intra-familial variation of fitness outcomes of dispersal has been less documented mostly linked to the difficulty to follow individuals beyond dispersal. Some studies found difference in short-term fitness measures: for instance in Gray jays (*Perisoreus canadensis)* winter survival was higher among philopatric dominant siblings who expelled their subordinate siblings from the natal territory^22^ or among Siberian jays (*Perisoreus infaustus),* where dominant siblings dispersed later than their subordinate siblings and experienced a higher breeding probability once they dispersed^9^*.*

Humans present an interesting model to study fitness consequences of dispersal along with intra-familial dynamics, because several differently-aged offspring often live together during childhood and beyond sexual maturity, and dispersal patterns strongly depend on sibling interactions^23,24^. These patterns are often driven by differences in access to family resources (e.g. linked with inheritance system or parental favouritism) according to birth order, sex or total number of siblings^23,25–27^. Moreover, consistent behavioural differences between siblings according to birth order have been highlighted^28,29^, thereby suggesting that siblings could have different fitness maximizing strategies. Given that datasets covering family structures throughout time with detailed records on dispersal behaviour are now available in humans, they offer a good opportunity to investigate whether fitness outcomes of dispersal differ between siblings. However, previous studies have focused mainly on dispersal determinants but did not investigate the fitness consequences of dispersal nor included any information on intra-familial competition over family resources^30,31^.

In this study, we used a large demographic dataset from preindustrial Finland to test whether the fitness outcomes of dispersal versus philopatry depend on the level of intra-familial competition. This dataset provides a high follow-up success of both philopatric and dispersing siblings, with complete record of the family structure. Moreover, the effect of sibling interactions on dispersal patterns and fitness outcomes in this population has been separately documented: the number of co-resident same-sex elder siblings is linked to a higher dispersal propensity^26^ and a lower lifetime reproductive success^24^. Among males, this effect was mainly driven by a competition over inheritable land resources with the eldest son inheriting most of parental resources and subsequently having an increased marriage probability, reproductive success and lower dispersal rates^24,26^. On the contrary, among females, competition between sisters was mainly mediated by competition over mating opportunities with elder sisters having a higher marriage probability and reproductive success. Furthermore, younger sisters were more likely to marry a landless man, which was linked to a higher dispersing probability after marriage^26^. Therefore, differences between siblings of different intra-sex birth rank are likely to be observed in the fitness outcomes of dispersal behaviour.

Specifically, we investigated the effect of intra-sex birth rank and dispersal strategy on three key indicators of lifetime reproductive success: probability of reproducing, lifetime fecundity, and offspring survival. As explained above, formulating clear hypotheses on the outcomes of dispersal specific to each sibling is difficult due to the fact that intra-familial configuration is not included explicitly in theoretical models and rarely in empirical studies. Therefore, in this study we combine the empirical results on the differences of dispersal patterns between siblings and investigated the two sides by which an individual can maximise its fitness: i.e. directly through its own reproductive success or indirectly through the reproductive success of its kin. First, under the hypothesis that dispersal phenotype is mainly a process to increase an individual’s direct fitness according to the level of intra-sex sibling competition, we predicted that philopatry benefited individuals with few or no same-sex elder siblings, whereas dispersal benefited those with more same-sex elder siblings. That-is-to-say, laterborn siblings should have a higher fitness when dispersing than remaining philopatric (e.g. by reducing the negative effect of co-resident same-sex elder siblings), whereas earlier-born siblings should have a lower fitness when dispersing than remaining philopatric (e.g. due to the loss of access to parental resources following their dispersal). Secondly, we investigated whether siblings’ dispersal could benefit philopatric kin, in order to assess the indirect fitness benefits of dispersal. Specifically, we tested whether the number of dispersing and philopatric same-sex younger siblings influenced the reproductive success of philopatric firstborns. To our knowledge, this is among the first studies showing how dispersal affects individual fitness outcomes compared to non-dispersing individuals with similar level of within-family competition in humans.

## Results

### Direct fitness outcomes

#### Males

First, we tested the prediction that dispersal could be a strategy to avoid costs of sibling interactions on personal reproductive success. Of males surviving to age 15 (*N* = 4485), 12.1% dispersed out of their natal parish (on average 51.8 km ± 3.1SE and at 26.9 years ± 0.5SE) (Table 1). 40% of dispersing males had at least one elder brother alive at 15, compared to only 7% of non-dispersing males. Despite these differences in sibling configuration between males dispersing and not dispersing, the hypothesis that dispersal decisions could have different fitness consequences for individuals with different numbers of same-sex elder siblings was not strongly supported by our results on any fitness outcomes. These findings were not confounded by differential socio-economic status (SES) or overall level of within-family sibling competition resulting from differences in the total number of siblings, which were controlled for in all of the models.

**Table 1:** Descriptive statistics of the study population.

	Males			Females		
	Probability of reproducing (N = 4485, 69%)	Offspring number (N = 3105, 5.4 ± 0.1 SE)	Proportion of offspring surviving to 15 (N = 3061, 70%)	Probability of reproducing (N = 4529, 78%)	Offspring number (N = 3533, 5.1 ± 0.0 SE)	Proportion of offspring surviving to 15 (N = 3491, 67%)
Dispersal category						
No dispersal (< 10 km): N (%)	3947 (88%)	2710 (87.3%)	2675 (87%)	3805 (84%)	2936 (83%)	2909 (83%)
Short distance dispersal (10–60 km):						
N (%)	372 (8%)	294 (9.5%)	288 (10%)	582 (13%)	501 (14%)	492 (14%)
Dispersal age (years) Mean ± SE	26.7 ± 0.7	27.1 ± 0.7	27.2 ± 0.8	26.3 ± 0.5	25.6 ± 0.6	25.6 ± 0.6
Dispersal distance (km) Mean ± SE	27 ± 0.6	27 ± 0.7	26.5 ± 0.7	28 ± 0.5	27 ± 0.5	28 ± 0.5
Long distance dispersal (> 60 km):						
N (%)	166 (4%)	101 (3.2%)	98 (3%)	142 (3%)	96 (3%)	90 (3%)
Dispersal age (years) Mean ± SE	27.3 ± 0.9	26.5 ± 1.1	26.7 ± 1.1	28.3 ± 1.2	28.7 ± 1.6	28.2 ± 1.6
Dispersal distance (km) Mean ± SE	146 ± 9.9	145 ± 11.1	146 ± 11.4	137 ± 8.6	136 ± 10.7	136 ± 10.9
Siblings						
Nb same-sex elder siblings Mean ± SE	0.77 ± 0.01	0.73 ± 0.01	0.7 ± 0.0	0.8 ± 0.02	0.8 ± 0.0	0.8 ± 0.0
Total nb of siblings Mean ± SE	4.0 ± 0.03	4.0 ± 0.04	3.9 ± 0.0	4.0 ± 0.03	3.9 ± 0.0	3.9 ± 0.0

**Figure 1:**
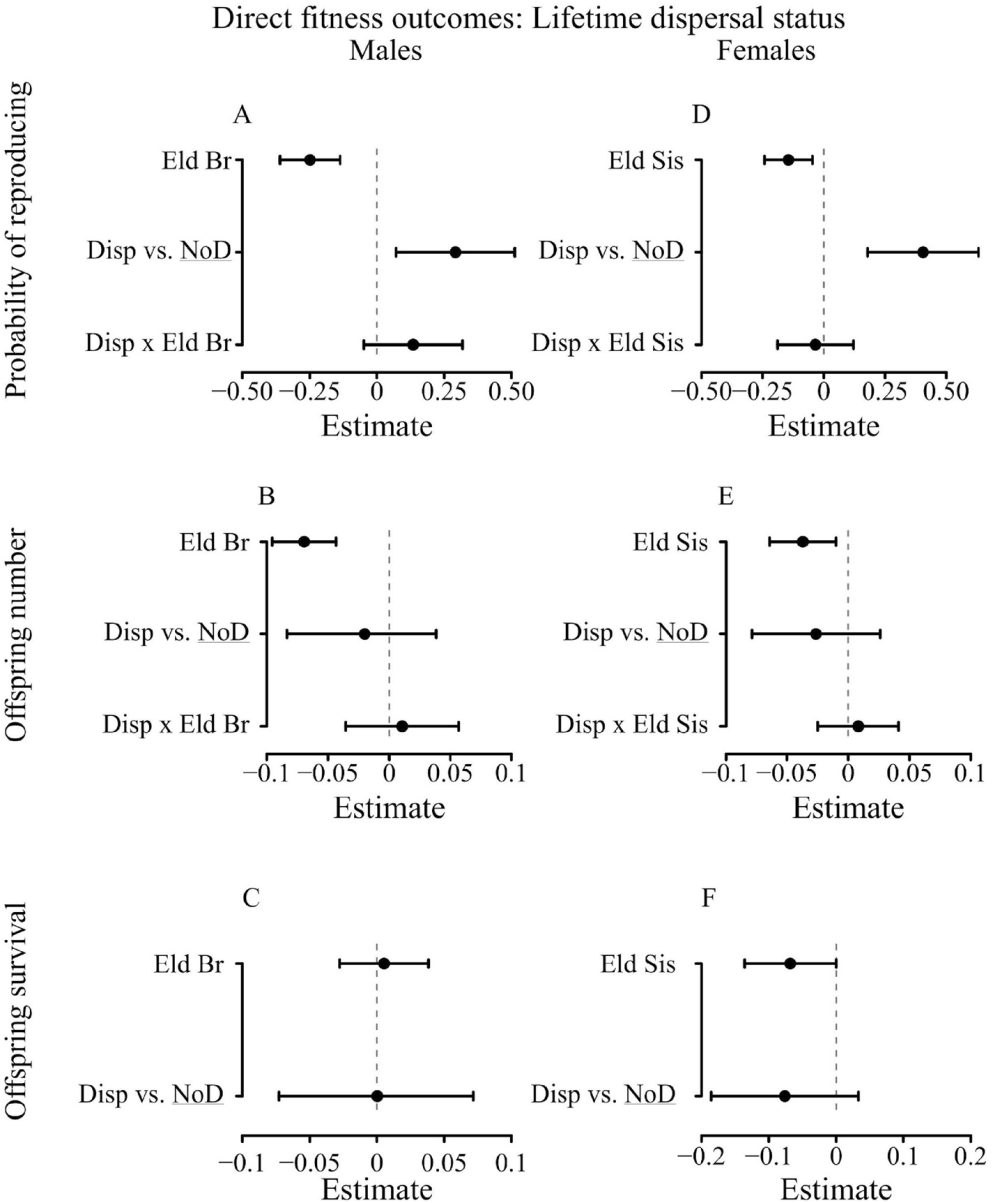
Lifetime dispersal status and reproductive success (direct fitness). Figures represent the averaged estimates and their 95% confidence intervals of the best models (full averaging) (error bars) for males’ (**A**) probability of reproducing (*N* = 4485) (Table S1), (**B**) offspring number (*N* = 3105) (Table S2), (**C**) offspring survival to age 15 (*N* = 3061) (Table S3); and for females (**D**) probability of reproducing (*N* = 4529) (Tables S1), (**E**) offspring number (*N* = 3533) (Tables S2, (**F**) offspring survival to age 15 (*N* = 3491) (Table S3). Figures represent the model averaged estimates and their 95% confidence intervals (error bars) of the factors (1) with an increment of 1 for the effect of elder brothers (Eld Br) and elder sisters (Eld Sis) (2) compared to the reference category (NoD = No dispersal) (underlined) for the effect of lifetime dispersal status. “x” denotes an interaction between variables and “Disp” stands for dispersed. All models controlled for birth parish, birth year, mother's identity, family SES, the number of opposite-sex elder siblings and the total number of siblings. Details of the models are available in the Methods and Supplementary information.

**Figure 2:**
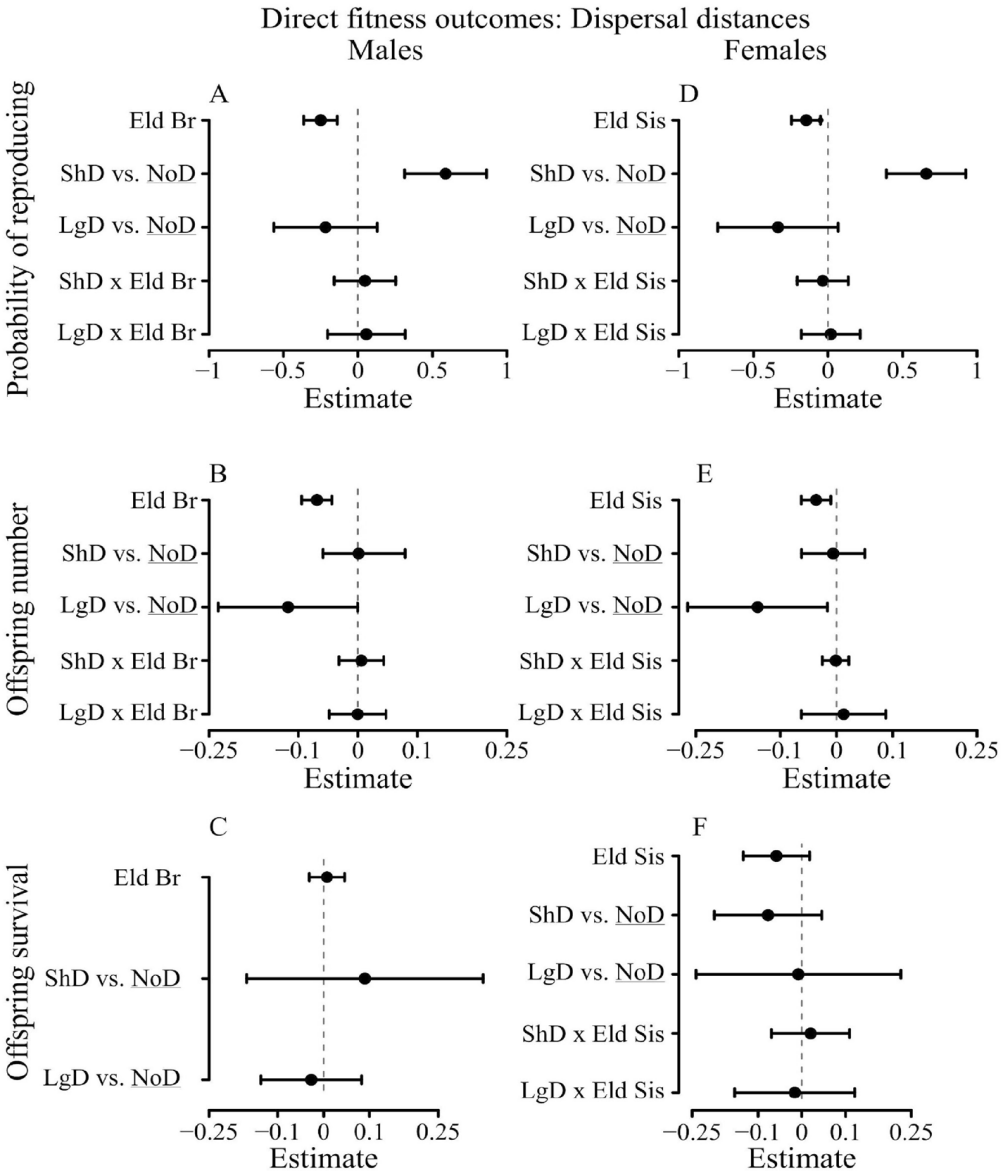
Dispersal distance and reproductive success (direct fitness). Figures represent the averaged estimates and their 95% confidence intervals (error bars) of the best models (full averaging) of the best models of the model selection for males’ (**A**) probability of reproducing (*N* = 4485) (Table S4), (**B**) offspring number (*N* = 3105) (Table S5), (**C**) offspring survival to age 15 (*N* = 3061) (Table S6); and for females (**D**) probability of reproducing (*N* = 4529) (Table S4), (**E**) offspring number (*N* = 3533) (Table S5), (**F**) offspring survival to age 15 (*N* = 4491) (Table S6). Figures represent the model averaged estimates and their 95% confidence intervals (error bars) of the factors (1) with an increment of 1 for the effect of elder brothers (Eld Br) and elder sisters (Eld Sis) (2) compared to the reference category (NoD = No dispersal) (underlined) for the effect of lifetime dispersal status. “x” denotes an interaction between variables, “ShD” stands for short distance dispersal (< 60 km) and “LgD” for long distance dispersal (> 60 km). All models controlled for birth parish, birth year, mother's identity, family SES, the number of opposite-sex elder siblings and the total number of siblings. Details of the models are available in the Methods and Supplementary Information.

First, 69% of males surviving to sexual maturity ever reproduced in their lifetime, but the presence of elder brothers decreased their probability of reproducing (β = 0.25; CI 95% [−0.36; −0.13]) (Table S1, Fig. 1, Figure S1). Although dispersal was overall linked to a higher probability of reproducing compared to philopatry (β = 0.29; CI 95% [0.06; 0.51]), it is unlikely that the negative effect of the presence of elder brothers on the probability of reproducing strongly differed between individuals who did or did not disperse as evidenced by the low statistical support for the interaction between the number of elder brothers and dispersal status. Specifically, (1) although the interaction was present among two of the best models selected, the model selection was uncertain (4 best models selected, out of the 6 tested, with a relative weight w_*i*_ ranging from 0.46 to 0.07) (Table 2); (2) the model-averaged estimates of the effect of the interaction and its 95% interval overlapped 0 (β = 0.10; CI 95% [−0.13; 0.34]). Results on dispersal distances (*N* = 4485, Fig. 2) were similar to models fitting dispersal as a binary variable. Indeed, we found little support for the effect of an interaction between dispersal distance category and the negative effect of elder brothers (Tables 3, S4) as the weight w_*i*_ of the model including the interaction between dispersal and the number of elder brothers was only of 0.15 and the model estimates for differences in the effect of elder brothers for different dispersal categories all overlapped 0. It indicates that dispersing men with many elder brothers were unlikely to enjoy a disproportional reproductive benefit compared to dispersing men with lower within-family competition. Moreover, the increased probability of reproducing among dispersing males was mainly driven by short distance dispersal (< 60 km, 69% of dispersing males): short distance dispersers had a higher probability of reproducing than philopatric males (β = 0.59; CI 95% [0.31; 0.86]), whereas we found no differences between males dispersing long distances and philopatric males (β = −0.22; CI 95% [−0.57; 0.13]).

**Table 2:** Summary of the best 95% a priori models with dispersal status fitted as a binary variable (dispersing vs. non dispersing) on (1) the probability of reproducing ; (2) lifetime fertility for individuals reproducing at least once ; (3) the proportion of children born surviving to age 15 for individuals for those at least one child had been followed to death or sexual maturity, including the total number of estimated parameters (K), the log-likelihood (LogLik), AIC differences relative to the minimum value in the set (*Δ*AIC) and the Akaike weight (*w*_*i*_).  “Eld Br”  stands for elder brothers, “Eld Sis” for elder sisters, “Disp Status” for lifetime dispersal status and “Fam SES” for the Socio-Economic Status of the family. See Methods for details on each analysis and the associated set of candidate models and Supplementary Information for the averaged estimates of the factors for the best fitting models (Tables S1–S3).

(1) Probability of reproducing					(2) Lifetime fertility (offspring number)					(3) Offspring survival				
Models	K	Log Lik	*Δ*AIC	*w*_*i*_	Models	K	Log Lik	*Δ*AIC	*w*_*i*_	Models	K	Log Lik	*Δ*AIC	*w*_*i*_
(A) Males (*N* = 4485)					(A) Males (*N* = 3105)					(A) Males (*N* = 3061)				
Full + Fam SES × Eld Br + Disp Stat × Eld Br	11	−2697.48	0.00	0.46	Full	10	−7660.89	0.00	0.46	Null	4	−4319.68	0.00	0.66
Full + Fam SES × Eld Br	10	−2698.75	0.52	0.36	Full + Disp Status × Eld Br	11	−7660.44	1.11	0.26	Full	9	−4316.35	3.39	0.12
Full + Disp Status × Eld Br	10	−2699.92	2.86	0.11	Full + Fam SES × Eld Br	11	−7660.83	1.89	0.18	Control	8	−4317.42	3.51	0.11
Full	9	−2701.32	3.65	0.07	Full + Fam SES × Eld Br + Disp Status × Eld Br	12	−7660.39	3.03	0.10	Full + Fam SES × Eld Br	10	−4315.51	3.72	0.10
(B) Females (*N* = 4529)					(B) Females (*N* = 3533)					(B) Females (*N* = 3491)				
Full	9	−2335.63	0	0.47	Full	10	−8565.64	0	0.45	Full + Fam SES × Eld Sis	10	−4817.78	0	0.53
Full + Fam SES × Eld Sis	10	−2335.25	1.34	0.25	Full + Fam SES × Eld Sis	11	−8565.13	0.99	0.28	Full + Fam SES × Eld Sis + Disp Status × Eld Sis	11	−4817.08	0.7	0.39
Full + Disp Status × Eld Sis	10	−2335.60	1.92	0.18	Full + Disp Status × Eld Sis	11	−8565.63	1.99	0.17	Null	4	−4825.76	3.7	0.08
Full + Fam SES × Eld Sis + Disp Status × Eld Sis	11	−2335.21	3.24	0.10	Full + Fam SES × Eld Sis + Disp Status × Eld Sis	12	−8565.13	3.01	0.10

**Table 3:** Summary of the best 95% a priori models with a difference in a range of Δ_i_ = 4 with dispersal status fitted as a 3-level categorical variable on (1) the probability of reproducing ; (2) lifetime fertility for individuals reproducing at least once ; (3) the proportion of children born surviving to age 15 for individuals for those at least one child had been followed to death or sexual maturity, including the total number of estimated parameters (K), the log-likelihood (LogLik), AIC differences relative to the minimum value in the set (*Δ*AIC) and the Akaike weight (*w*_*i*_). “Eld Br” stands for elder brothers, “Eld Sis” for elder sisters, “Disp Cat” for the dispersal distance category and “Fam SES” for the Socio-Economic Status of the family. See Methods for details of each analysis and the associated set of candidate models and Supplementary Information for the averaged estimates of the factors for the best fitting models (Tables S4-S6).

(1) Probability of reproducing					(2) Lifetime fertility (offspring number)					(3) Offspring survival				
Models	K	Log Lik	*Δ*AIC	*w*_*i*_	Models	K	Log Lik	*Δ*AIC	*w*_*i*_	Models	K	Log Lik	*Δ*AIC	*w*_*i*_
(A) Males (*N* = 4485)					(A) Males (*N* = 3105)					(A) Males (*N* = 3061)				
Full + Fam SES × Eld Br	11	−2692.03	0.00	0.61	Full	11	−7659.06	0.00	0.61	Null	4	−4319.68	0.00	0.54
Full + Fam SES × Eld Br + Disp Cat × Eld Br	13	−2690.78	1.51	0.28	Full + Fam SES × Eld Br	12	−7659.00	1.90	0.24	Full	10	−4314.82	2.34	0.17
Full	10	−2694.75	3.43	0.11	Full + Disp Cat × Eld Br	13	−7658.45	2.81	0.15	Control	9	−4315.92	2.52	0.15
										Full + Fam SES × Eld Br	11	−4313.97	2.65	0.14
(B) Females (*N* = 4529)					(B) Females (*N* = 3533)					(B) Females (*N* = 3491)				
Full	10	−2327.60	0.79	0.59	Full	11	−8563.70	0.00	0.47	Full + Fam SES × Eld Sis	11	−4817.62	0.00	0.51
Full + Fam SES × Eld Sis	11	−2327.53	1.88	0.23	Full + Fam SES × Eld Sis	12	−8563.20	1.01	0.29	Full + Fam SES × Eld Sis + Disp Status × Eld Sis	13	−4816.40	1.59	0.23
Full + Disp Cat × Eld Sis	12	−2326.80	2.44	0.18	Full + Disp Status × Eld Sis	13	−8562.85	2.33	0.15	Null	4	−4825.76	2.21	0.17
					Full + Fam SES × Eld Sis + Disp × Eld Sis	14	−8562.31	3.26	0.09	Full	9	−4821.31	3.36	0.10

Second, the negative effect of elder brothers on lifetime fertility (5.4 ± 0.1SE) among males reproducing at least once (*N* = 3105; β = −0.07; CI 95% [−0.10; −0.04]) was unlikely to drastically differ between males dispersing and not dispersing (Tables 2, S2, Fig. 1): (1) the model including the interaction between elder brothers and dispersal status received a low support (w_*i*_ = 0.26); (2) the model-averaged estimate of the interaction overlapped zero (β = 0.03; CI 95% [−0.04;0.06]). Overall, the number of children did not depend on a male’s dispersal status (β = −0.02; CI 95% [−0.08; 0.04]). Similarly, among models on dispersal distances, little evidence of variation of the outcomes of dispersal according to intra-sex birth-order was found: (1) the model ranked as the best model was the full model (w_i_ = 0.61), whereas the model containing the interaction between dispersal and the presence of elder brothers only received a support of 0.15; (2) the negative effect of the presence of elder brothers (β = −0.07; CI 95% [−0.09; −0.04]) (Tables 3, S5, Fig. 2) among non-dispersers was similar among males dispersing short distances (β = −0.00; CI 95% [−0.05; 0.05]) and among those dispersing long distances (β = 0.01; CI 95% [−0.03; 0.04]) as both confidence intervals of the estimates overlapped zero.

Finally, child survival to age 15 (70% of all children born) was not strongly linked to any of the variables considered: (1) among the 4 models selected in the best model set (w_*i*_ ranging from 0.10 to 0.66) the null model obtained higher weight (w_*i*_ = 0.66) (2) all model-averaged estimates overlapped zero (Tables 2, S3, Fig. 1). It suggests that neither elder brothers nor lifetime dispersal status had a strong effect on the survivorship of a male’s children. Results of model on dispersal distances were similar (Tables 3, S6, Fig. 2).

#### Females

Of females surviving to age 15 (*N* = 4529), 16.1% dispersed on average at 26.7 years (± 0.5SE) and 44.1 km (± 2.0SE) away from the natal parish (Table 1). At age 15, 40% of the dispersing females had at least one elder sister alive compared to only 9% of non-dispersing females. Similarly to males, our results offer little support to the hypothesis that the fitness outcomes of lifetime dispersal status were linked to the level of intra-sex sibling competition in the natal parish. All the models controlled for the family SES and the total number of siblings.

First, 78% of females surviving to sexual maturity reproduced. Although females dispersing were overall more likely to reproduce than philopatric females and having elder sisters reduced a female’s probability of reproducing (β = 0.41; CI 95% [0.18; 0.63]) (Table S1*,* Fig. 1, Figure S1), the negative effect of elder sisters on probability of reproducing did not depend on whether they dispersed or not. Specifically, (1) the model containing the interaction between elder sisters and dispersal status received a low support (w_*i*_ = 0.18) (Table 2); (2) the negative effect of the presence of elder sisters among females not dispersing (β = −0.15; CI 95% [−0.24;−0.05]) was not different among dispersing females (estimates of the interaction: β = −0.03; CI 95% [−0.18; 0.12]). Similarly, models including dispersal distances did not provide any evidence that the outcomes of dispersal phenotype might vary between sisters (Tables 3, S4, Fig. 2): (1) the model containing the interaction between dispersal status and elder sisters’ presence received a low support (w_*i*_ = 0.18); (2) estimates of the effect of the interaction overlapped zero (interactions between elder sisters and long distance dispersal: β = 0.01; CI 95% [−0.17.;0.13]; and short distance dispersal: β = −0.03; CI 95% [−0.16; 0.19]). Finally, the higher probability of reproducing among dispersing females was likely to be mainly driven by short distance dispersers (80% of females dispersing): females dispersing at short distances were more likely to reproduce than philopatric females (β = 0.66; CI 95% [0.39; 0.92]), but those dispersing further away were not (β = −0.33; CI 95% [−0.74; 0.07]).

Second, elder sisters had a negative effect on lifetime fertility (5.1 ± 0.05SE, β = -0.04; CI 95% = [−0.06;−0.01]) among females reproducing at least once (*N* = 3533). However, we found overall no difference between offspring number born to dispersing and non-dispersing females (β = −0.03; CI 95% [−0.08; 0.02], Table S2, Fig. 1) and the negative effect of the presence of elder sisters did not depend on dispersal status (β = 0.00; CI 95% [−0.03; 0.03], Table S2, Fig. 1) as the best approximating model (w_*i*_ = 0.45) included only the main effect of elder sisters (Table 2), with the model including the interaction between the number of elder sisters and dispersal status receiving a much lower support (w_*i*_ = 0.17). We obtained similar results when dispersal distance was included (Tables 3, S5*,* Fig. 2). Like males, females dispersing at least 60 km away from their natal parish (long-distance dispersal) achieved a lower lifetime fertility than non-dispersing females (β = −0.14; CI 95% [−0.27; −0.02]), whereas we did not find such differences between females not dispersing and dispersing at short distances (β = −0.01; CI 95% [−0.06; 0.05]). However, the effect of the presence of elder sisters was found to be similar between females not dispersing and those dispersing long distances (β = 0.02; CI 95% [−0.07; 0.10]) or short distances (β = −0.00; CI 95% [−0.03; 0.03]).

Finally, whether women with intense vs. low within-family competition dispersed or not did not influence the proportion of their offspring successfully raised to adulthood (67% of a woman’s children). The best model did not contain an interaction between dispersal status and number of elder sisters (w_*i*_ = 0.53; Table 2), with the interaction only present in the second best model (w_*i*_ = 0.39). Furthermore, the estimates of the negative effect of elder sisters (β = −0.07; CI 95% [−0.14; 0.00]) did not strongly differ among dispersing females (β = 0.06; CI 95% [−0.07; 0.16], Table S3, Fig. 1). When considering models including dispersal distances, none of the variables included in the models were associated with offspring survival as all model-averaged estimates overlapped 0 (Tables 3, S6, Fig. 2).

Consequently, overall, we found no evidence that by dispersing, either males or females would have alleviated the negative effects of sibling competition on their personal reproductive success.

### Indirect fitness outcomes

#### Males

Second, we tested whether dispersal could be a strategy to avoid costs of sibling competition on reproductive success of the remaining, philopatric offspring, and thus a way to increase one’s fitness indirectly through the success of the elder siblings. Philopatric elder brothers (*N* = 1282) had about 75% of all their younger brothers remaining philopatric during lifetime. Overall, there was no evidence that dispersal could provide younger brothers any indirect fitness benefits through an increase of their eldest brother’s reproductive success. Instead, of the three sets of candidate models on outcomes of reproductive success considered, the model containing the effect of the number of philopatric younger siblings was only retained once among the best candidate model set, namely in models on lifetime fertility (Tables 4, S7–S9). However, the model-averaged estimates of this latter variable overlapped zero thereby indicating that its effect was weak (β = −0.00; CI 95% [−0.03; 0.02]). All the models controlled for the family SES and the total number of siblings.

**Table 4:** Indirect fitness benefits. Summary of the best 95% a priori models with a difference in a range of Δ_i_ = 4 for non-dispersing firstborns of each sex on (1) the probability of reproducing ; (2) lifetime fertility for individuals reproducing at least once ; (3) the proportion of children born surviving to age 15 for individuals for those at least one child had been followed to death or sexual maturity, including the total number of estimated parameters (K), the log-likelihood (LogLik), AIC differences relative to the minimum value in the set (*Δ*AIC) and the Akaike weight (*w*_*i*_). See Methods for details of each analysis and the associated set of candidate models and Supplementary Information for the averaged estimates of the factors for the best fitting models. See Methods and Supplementary Information for details (Tables S7–S9).

(1) Probability of reproducing					(2) Lifetime fertility (offpsring number)						(3) Offspring survival					
Models	K	Log Lik	*Δ*AIC	*w*_*i*_	Models	K	Log Lik	*Δ*AIC	*w*_*i*_		Models	K	Log Lik	*Δ*AIC	*w*_*i*_	
(A) Males (*N* = 1282)					(A) Males (*N* = 922)						(A) Males (*N* = 911)					
Control	6	−738.89	0.00	1	Control	7	−2339.89	0.00	0.85		Null	4	−1448.51	0.00	0.81	
					Full	11	−2337.56	3.51	0.15		Control	6	−1447.96	2.94	0.19	
(B) Females (*N* = 1083)					(B) Females (*N* = 835)						(B) Females (*N* = 827)					
Control	6	−576.15	0 .00	0.84	Null	5	−2060.88	0.00	0.59		Null	4	−1249.53	0.00	0.86	
Null	4	−579.83	3.32	0.16	Control	7	−2059.22	0.58	0.41		Control	6	−1249.34	3.67	0.14	

#### Females

A philopatric elder sister (*N* = 1083) had about 74% of her total number of younger sisters remaining philopatric during lifetime. Like for males, there was no evidence that departure of younger sisters provided any fitness benefits to their eldest sister. Indeed, results of the model selection on all outcomes of reproductive success never retained “Number of philopatric younger sisters” in the best candidate model set (Tables 4, S7–S9) thereby indicating that including it never improved model fit enough: the dispersal behavior of a female’s younger sisters had thus probably a very weak influence on her own reproductive success. Consequently, both the relatively low dispersal rate and the results of the models suggest that it was unlikely that siblings dispersed purely to promote the reproductive success of their remaining philopatric siblings through reduced sibling competition. All the models controlled for the family SES and the total number of siblings.

## Discussion

Determining the fitness consequences of dispersal and its interplay with sibling interactions is important for understanding the evolution of dispersal and family living and could shed light both on dispersal drivers and on variation of fitness maximizing strategies between family members. Using a large demographic dataset from pre-industrial Finland, we tested whether the outcomes of dispersal decisions on reproductive success depended on an individual’s intra-sex birth rank. Overall, our findings did not strongly support this hypothesis (Table 5). Instead, the fitness outcomes of dispersal status were similar regardless of an individual’s number of same-sex elder siblings, despite the fact that within-family competition with such same-sex siblings significantly reduced reproductive success. As the model selection was uncertain on most of the fitness outcomes considered, it is however not possible to draw strong conclusions on the possible variation of the within-family competition with dispersal status, but rather that altogether, the support for this hypothesis was overall low. Furthermore, we also tested whether dispersal could benefit individuals indirectly (e.g. through a decrease of local sibling competition), but found no support for such an effect. Our results are unlikely to be confounded by the family SES or its interaction with the number of same-sex elder siblings as these effects were all controlled for in our analyses. These findings do not support the investigated hypotheses that between-individual differences in dispersal behaviour of family members could be a strategy to avoid the negative consequences of sibling interactions either on personal reproductive success or that of the remaining, philopatric siblings in the natal territory in this population. However, as our study is not experimental, it is not possible to strongly conclude on the relative costs and benefits of each strategy.

**Table 5:** General summary of the hypotheses tested and the results of the models investigating direct and indirect fitness benfits of dispersal on each sex on: (1) the probability of reproducing ; (2) lifetime fertility for individuals reproducing at least once ; (3) the proportion of children born surviving to age 15 for individuals for those at least one child had been followed to death or sexual maturity., “*w*_*i*_” stands for Akaike weight,"Disp" stands for “dispersing”, “Eld Br” for elder brothers, “Eld Sis” for elder sisters, “Y Br” for younger brothers, “Y Sis” for younger sisters and “Disp Cat” for dispersal category. See Methods for details of each analysis and the associated set of candidate models and Tables 2–4 for results of the model selection and Supplementary Information for the averaged estimates of the factors for the best fitting models.

	Variable	Prediction if hypothesis supported	(1) Probability of reproducing	(2) Lifetime fertility	(3)Offspring survival
Direct fitness benefits: dispersal fitted as a binary variable					
Male**s**	Best 95% models	w_i_ of the model(s) containing the interaction = 1	w_i_ = 0.57	w_i_ = 0.26	Not present
	Disp Cat × Eld Br	Positive estimate	Overlaps 0	Overlaps 0	
	Hypothesis tested	Variation of the effect elder brothers with dispersal status?	Low support	Very low support	No support
Females	Best 95% models	Wi of the model(s) containing the interaction = 1	w_i_ = 0.28	w_i_ = 0.27	w_i_ = 0.39
	Disp Cat × Eld Br	Positive	Overlaps 0	Overlaps 0	Overlaps 0
	Hypothesis tested	Variation of the effect elder sisters with dispersal status?	Very low support	Very low support	Very low support
Direct fitness benetits: Dispersal fitted as 3 level categorical variable					
Males	Best 95% models	w_*i*_ of the model(s) containing the interaction = 1	w_*i*_ = 0.28	w_*i*_ = 0.15	Not present
	Disp Cat × Eld Sis	Positive estimate	Overlaps 0	Overlaps 0	
	Hypothesis tested	Variation of the effect elder brothers with dispersal status?	Very low support	Very low support	No support
Females	Best 95% models	w_*i*_ of the model(s) containing the interaction = 1	w_*i*_ = 0.18	w_*i*_ = 0.24	w_i_ = 0.23
	Disp Cat × Eld Sis	Positive estimate	Overlaps 0	Overlaps 0	Overlaps 0
	Hypothesis tested	Variation of the effect elder sisters with dispersal status?	Very low support	Very low support	Very low support
Indirect fitness effects					
Males	Best 95% models	Full model containing the dispersal status of younger brothers	Not present	w_*i*_ = 0.85	Not present
	Nb non disp Y Br	Negative estimate		Overlaps 0	
	Prop disp Y Br	Positive estimate		Overlaps 0	
	Hypothesis	Dispersing younger brothers is linked to higher fitness for non dispersing elder brothers	No support	Very low support	No support
Females	Best 95% models	Full model containing the dispersal status of younger sisters	Not present	Not present	Not present
	Nb non disp Y Sis	Negative estimate			
	Prop disp Y Sis	Positive estimate			
	Hypothesis	Dispersing younger sisters linked to higher fitness for non dispersing same-sex elder sisters	No support	No support	No support

Interpreting in detail the rate of dispersal and the fitness outcomes of dispersal per se is beyond the scope of this work, as similar in-depth studies among other human populations are currently lacking. In our population, about 14% of individuals dispersed from their natal parish, which was slightly lower than the rates of dispersal found in other Western historical population (16% in historical Germany^23^ and 36% in historical Sweden^25^), but consistent with genetic studies implying a long population history of small breeding units, little gene flow and isolation of local populations by density^32^. Furthermore, as this is the first study to investigate the fitness outcomes of dispersal in humans, we limit our interpretation to the possible drivers of the effects highlighted and to examine the outcomes of dispersal of individuals relative to other subgroups within our population. Dispersal could modify reproductive success through different underlying mechanisms depending on the variable studied. In humans, the probability of reproducing is closely linked to access to mating opportunities, and the lifetime number of children correlates strongly with the age at the start of reproduction^33^, whereas offspring survival correlates with access to resources (e.g. SES or family support). As dispersal status per se was not strongly linked with offspring number or offspring survival, the effect of dispersal on the timing of reproduction or overall access to resources was probably very weak.

On the contrary, dispersers enjoyed a higher probability of reproducing than philopatric individuals, thereby suggesting that dispersal was somehow linked to an increased access to mating opportunities and that dispersal per se was beneficial. However, most individuals remained philopatric (86%), which seems paradoxical. Similarly to other historical populations^34^, historical Finns often left the parental household at the time of marriage, or a few years after^35^. However, departure after marriage cannot be the sole explanation for the higher probability of reproducing among dispersers, as a considerable proportion of dispersers who managed to marry in their lifetime left while still unmarried (44% of males and 39% of females).

Furthermore, our results could be driven by sex-specific (e.g. through relieve of same-sex sibling competition) and non-sex specific mechanisms. As the fitness outcomes of dispersal are qualitatively similar for both sexes, mediating factors common to both sexes are likely to play a stronger role than sex-specific factors. Several non-sex specific factors could be involved here. First, as dispersal in humans is condition-dependent, individuals might only leave when an opportunity is available (e.g. for a marriage or a job)^31^. In the absence of any opportunity, individuals would otherwise stay in the family household because costs of dispersal might be too high. However, evaluating such costs from our data is not possible, as information on an individual’s potential opportunities or local ecological conditions before dispersal is rarely available^36^. Variation in opportunity for dispersal could explain why we detected no dispersal costs apart from the lower lifetime fertility of long distance dispersers. Further studies are therefore needed to estimate the relative success of dispersers with good opportunities and those not having any better prospect, but quantitative data on this is rarely available for any species. Estimations of the real costs of dispersal is extremely difficult as it often requires the experimental manipulation of dispersal phenotype^19,37,38^. As in this study, we use empirical data, this study has to rely on the assumption that some individuals do not behave optimally, to be able to highlight fitness differences between dispersing strategies. Furthermore, dispersal conditional on the availability of opportunities would have consequences on population structure^39^. Indeed, the philopatric population would be both constituted of individuals not migrating because it is the best choice for them, and of individuals who cannot afford dispersing. On the contrary, among dispersers there would be only individuals dispersing to maximize their fitness^18^. Such interpretations are consistent with results in an American historical population (USA)^40^ or other non-human species, e.g. see^41^. Second, the higher success of dispersers could suggest that kin network had a strong role in this population. For instance, kin could facilitate obtaining information about opportunities outside the natal territory or provide support after dispersal. It could especially explain why only short distances dispersers have higher probabilities of reproducing compared to long distance dispersers. This result could be compared to studies on non-human species, where dispersers strongly rely on information of the quality of the new territory^21,42^ or the existence of coalition of kin at dispersal^43,44^.

Our study focused on the existence of different dispersal strategies between family members, mediated here by sibling interactions. This hypothesis was not supported, as the outcomes of dispersal were similar across all siblings. An alternative explanation is that since we only considered intra-sex birth-order and defined dispersal as departure from the birth parish (intra-parish movements were not systematically recorded in our population), this might not be detailed enough to highlight differences of fitness maximizing strategies (e.g. information on personality). For instance, a previous study on dispersal in this population showed that most females were dispersing at the time of marriage whereas males were leaving mainly before marriage or synchronously with marriage^26^. It suggests the potential existence of a mix of dispersing strategies within the population, but due to data limitation, it was not possible to include this parameter. As suggested by Roulin and his collaborators^11^, siblings of similar birth rank might still occupy different niches in the family settings, but it might require more detailed behavioral data to be investigated empirically, or experimental studies manipulating dispersal behavior^41^. However, as fine patterns of other life-history traits have been highlighted in this population using the same definition of dispersal (such as variation of dispersal probability between birth ranks)^26^, it seems unlikely that strong differences in fitness maximizing strategies would have been entirely unnoticed. Therefore, the most likely explanation is that differences of dispersal patterns between birth ranks were not mediated by differences of fitness maximizing strategies. It is also unlikely that, similarly to some cooperative breeding species^45^, philopatry might provide indirect benefits for individuals experiencing strong negative effects of sibling competition. Indeed, both this study on effects on probability of reproducing and offspring number and a former one on offspring survival, failed to highlight such evidence in this population^46^. Overall, explaining patterns of dispersal and especially the higher proportion of later-borns dispersing by the maximization of an individual inclusive fitness therefore seems unlikely.

Finally, in the context of family conflicts, dispersal does not necessarily involve individual or sibling fitness benefits and could instead benefit other kin^4^. From a parental perspective, forcing or manipulating their offspring to disperse could benefit their own fitness, for example for their own reproduction^.^e.g.^47^. As dispersal is commonly phenotype dependent parents may also not enforce dispersal, but could only manipulate phenotype for instance through different access to food^48^. Similarly, investigating simultaneously the relative importance of different types of conflicts (e.g. parent–offspring conflict vs sibling competition) in the decision to disperse along with its outcomes of dispersal would enable a more accurate interpretation of the fitness outcomes of dispersal. More generally, control of dispersal decision is likely to be a key parameter in the understanding of dispersal. Indeed, Rodrigues and Gardner^49^ predicted variation in dispersal patterns depending on the relatedness (kin vs. nonkin) and the identity of individuals (mother vs. offspring) exerting control over the dispersal decision. In humans, evidence of biased parental investment between siblings and the variability of inheritance systems support the idea that parents might be the main beneficiaries of differential fitness maximizing strategies among siblings^49,50^.

Consequently, the interplay between dispersal and sibling interactions is likely to vary between populations with different inheritance, economic or family system (e.g. extended vs. joint families), as sibling interactions^51–53^ and dispersal patterns^23,54^ strongly depend on these factors. Unfortunately, in most family-living species, including humans, benefits of dispersal for different family members remain unclear as few data on behavioral mechanisms preceding dispersal exist and studies other than ours comparing the relative fitness outcomes of dispersal between kin are lacking, which precludes the understanding of dispersal in families and furthermore limits the generalization of our results to other human populations.

To conclude, despite the extensive literature on the importance of kin interactions in dispersal behaviour and the potential variation of fitness maximizing strategies, studies testing the fitness outcomes of dispersal according to intra-familial interactions are lacking. We present here the first study documenting the fitness outcomes of dispersal in humans. We show that kin differences in dispersal behaviour cannot be explained by variation in dispersal payoffs or by dispersal being driven by sibling competition avoidance (due to benefits either on personal reproductive success or on the remaining philopatric siblings). At a larger scale, our study therefore stresses the need for studying the relative outcomes of dispersal at the family level in order to understand dispersal behaviour and its evolution in family living species.

## Methods

### Study population

The demographic Finnish dataset used in this study was compiled from records of the Lutheran church which was obliged by law to document all births, marriages, deaths and movements between parishes in the whole country since 1749^55,56^. These data were digitized by the Genealogical Society of Finland HisKi project, and are available at: https://hiski.genealogia.fi/historia/indexe.htm. All methods were performed in accordance with the relevant guidelines and regulations. The study period was limited to individuals born before 1900, i.e. before the spread of industrialism^57^, the transition to reduced birth and mortality rates^58,59^, changes in kin networks^60^ and development of the Finnish railway^61^. These populations studied mainly depended on farming for their livelihood and were supplemented with fishing in the coastal areas. The standard of living was low with both famines and diseases being common^62,63^. We categorized all individuals into two family socio-economic status (SES) groups according to the father’s occupation: low (e.g. farmless families and servants, tenant farmers and fishermen) and high (e.g. landowners, shipmasters)^64^. The inheritance system usually favored the eldest son (primogeniture) and the predominant household was composed of the eldest son, his wife, their children, his parents and any unmarried sibling^65^. The mating system was monogamous, patrilocal and divorce was forbidden^66^. We expected a low paternity uncertainty as previous studies combining genetic and genealogical information estimated a low extra-paternity rate in Western historical populations (around 1%)^67^.

We only included individuals surviving to sexual maturity (age 15, the age of the youngest known reproducer in our population), followed at least up to the age where 90% of the individuals stopped reproducing (50 and 45 for males and females, respectively), for whom all the variables controlled for in our statistical analysis were available and whose mother’s full reproductive life was recorded, in order to have accurate information on sibship configuration. We excluded twins as their presence is associated with different family dynamics^68^. Dispersal was defined as departure from the birth parish as this information was systematically recorded in the parish registers at the time. Intra-parish movements were considered as non-dispersal, as this information was rarely recorded. We limited the study to the first movement of an individual out of his/her birth parish. Individuals dispersing during childhood (before age 15) as family members were excluded from the data set (< 1% of the overall sample). Using the geographical coordinates of the parishes, we calculated the distances of dispersal for individuals staying in Finland (i.e. not those going abroad, < 1% of the overall sample). As the geographical size of the parishes varied, considering only dispersal from natal parish during lifetime might overestimate dispersal in small parishes and underestimate it in larger parishes. To minimize this bias, individuals dispersing to a parish located below 10 km away from the birth parish were considered as non-dispersing. Moreover, travelling speed by foot based on the topography and geography of Finland estimated a travelling speed of an average of 3 km/h in Southern Finland^69^. Overall, about 14% of individuals dispersed from their natal parish, which was slightly lower than the rates of dispersal found in other Western historical population (16% in historical Germany^23^ and 36% in historical Sweden^25^), but consistent with genetic studies implying a long population history of small breeding units, little gene flow and isolation of local populations by density^32^. The final study sample contained 4485 focal males and 4529 focal females born 1720–1900 to 3716 mothers, in 104 geographically distinct parishes located in mainland or in South-Western coastal areas of Finland. All the analyses were separated by sex as dispersal patterns and sibling interactions strongly differ between sexes in this population^26,46^.

### Statistical analyses

#### Direct fitness outcomes

We analysed the effects of dispersal and intra-sex birth order on reproductive success. We fitted two sets of models depending on the lifetime dispersal status: (1) dispersal fitted as a binary variable (no dispersal vs. dispersal); (2) dispersal fitted as a 3-level categorical variable according to dispersal distance (*no dispersal*, *short distance dispersal* when dispersal distance was below 60 km, and *long distance dispersal* when dispersal distance was above 60 km) to investigate whether dispersal outcomes depended on the potential of remaining in contact with the philopatric kin. We investigated reproductive success at two steps because the large number of individuals having no children (26%) gave rise to a bi-modal distribution and prevented analysis of the number of children in a single model. We considered the following variables: (1) the probability of reproducing in an individual’s lifetime; (2) lifetime fecundity (i.e. the total number of offspring), among individuals reproducing at least once (3107 males and 3539 females); (3) offspring survival, measured as the proportion of an individual’s offspring successfully raised to age 15. This latter sample only included individuals for whom at least one child was followed until adulthood (3061 males and 3491 females). Because the fate of all offspring was not always known, and including only complete families might bias the sample, each individual was weighted with the proportion of his/her offspring that were successfully followed until 15 or their death.

*Probability of reproducing* was analysed with a binomial error structure and a logit link function. To take into account the overdispersion of the data, we analysed *lifetime fecundity* with a negative binomial error structure and a logarithm link function. Finally, we analysed *offspring survival to adulthood* by fitting the number of surviving offspring as a response term with a logit link function and a binomial denominator equal to lifetime fecundity. All statistical analyses were conducted in R 3.0.3^70^ using generalized linear mixed effects models (GLMMs) (packages *lme4* for binomial error structure and *glmmADMB* for negative binomial error structure)^71,72^.

##### Multi-model selection and model averaging techniques

We used AIC model selection techniques (R package *MuMIn*)^73^ and an a priori model set corresponding to the different interactions which could have an effect on fitness outcomes (namely the interactions between same-sex elder siblings and lifetime dispersal status, and between same-sex elder siblings and family SES) (detailed below). These models were ranked according to their goodness-of-fit to the data based on the Akaike Information Criterion (AIC)^74,75^. The difference in AIC *(Δi)* between the model with the lowest AIC (considered as the best model) and the other models provides a measure of how much more likely the best model is than model *i*. Following Symonds and Moussalli^75^, we only considered models with Δ*i* values up to 4. We computed model-averaged parameters and error estimates for each variable (full model averaging)^75,76^. We also calculated the odds ratios (OR) of the effects and the 95% confidence interval (CI 95%) for binary response variables. When the 95% confidence interval excludes one, the variable studied is considered as associated with the response variable. Conversely, when the 95% confidence interval includes one, it indicates that the variable is not strongly associated with higher or lower reproductive success, and therefore that its effect in our analyses is not found to be strong.

##### Candidate model set

We considered a set of 6 models for each reproductive success outcome and for each sex. We considered: (1) a null model, *Null*, including only the random terms (i.e. mother’s identity, birth year and birth parish, see details in the *Control variables section*); (2) a control model, *Control*, containing the random terms and the potentially confounding variables (see details in the *Confounding variables section*); (3) a full model, *Full*, including the effect of same-sex elder siblings, the dispersal status and the confounding variables (4) a full model including the interaction between dispersal status and the number of same sex elder siblings: *Full* + *Eld Br/Eld Sis *×* Disp Status,* testing the prediction that the effect of dispersal was different between earlier-borns and later borns; (5) a full model with the interaction between the number of same-sex elder siblings and the family SES: *Full* + *Eld Br/Eld Sis* × *Fam SES* testing the prediction that the effect of sibling competition differed between the different SES groups; finally (6) a model containing both of these interactions: *Full* + *Disp Status* × *Eld Br/Eld Sis* + *Eld Br/Eld Sis* × *Fam SES*. We fitted the effect of same-sex elder siblings as a continuous variable and it corresponded to the number of elder brothers for males or elder sisters for females, alive when an individual reached the age 15. As previous studies showed the effect of elder brothers on their younger brothers was best fitted as a binary variable (heir of the family vs. non-heir)^26^, we also ran the models for males with elder brothers fitted as a binary variable. As the results were qualitatively similar than those with elder brothers fitted as a continuous variable, they are not presented here (available upon request).

##### Control variables

The models controlled for the following variables as fixed factors: number of same-sex elder siblings, opposite-sex elder siblings and the total number of siblings (i.e. including younger siblings) alive when the focal individual turned 15 and family SES (2-level categorical variable). Numbers of elder brothers and elder sisters above 3 were pooled to avoid an excessive influence of extreme numbers. The total number of siblings and the number of elder brothers and elder sisters were centered and divided by 10. We included birth parish, birth year and the mother’s identity as random factors to take into account for the potential dependency of individuals born in the same parish, during the same time period or from the same mother.

#### Indirect fitness outcomes

We then investigated whether dispersal could provide indirect fitness benefits (e.g. mediated by a decrease of kin competition in the natal family). We restricted the sample to non-dispersing firstborns of each sex, as we hypothesize that they would be the most likely to benefit from the departure of their same-sex younger siblings. The final dataset contained 1282 males and 1083 females. Similarly to previous analyses, we fitted GLMMs on outcomes of reproductive success (*probability of reproducing*, *lifetime fecundity* and *offspring survival to adulthood*). Previous models on dispersal predicted that the most important determinant was the number of philopatric kin and not the number of dispersing ones^16,49,77^. Therefore, we included the variable “number of philopatric same-sex younger siblings” instead of the number of dispersing same-sex younger siblings. We also included a 4-level categorical variable (0–25%, 25–50%, 50–75% and 75–100%) on the proportion of same-sex younger siblings dispersing during their lifetime, in order to control for the total number of same-sex younger siblings. The control variables were the total number of siblings and the family SES. All variables concerning siblings only included those alive when the focal individual turned 15 and were centered and divided by 10. The random terms included were the same variables as in the previous analyses. Like the models on the direct fitness outcomes of dispersal, we used model averaging techniques and considered a set of 4 models: (1) a null model, *Null*, containing only the random terms (2) a control model, *Control*, containing the random terms and the potentially confounding variables (3) a full model, *Full*, comprising the number of philopatric same-sex younger siblings, the proportion of same-sex dispersing younger siblings and the confounding variables (4) a full model where the number of same-sex younger siblings was also fitted as a quadratic variable. Due to limitations of the sample size, we did not test for any interactions with the family SES.

## Supplementary Information

Supplementary Information.

## Data Availability

The datasets used in the analysis are available at 10.5281/zenodo.7855118.
