# Supplementary material for: Sibling competition, dispersal and fitness outcomes in humans
Source: Sci Rep. 2023 May 9;13:7539. doi: 10.1038/s41598-023-33700-3 (PMC10169773; doi:10.1038/s41598-023-33700-3)
Supplement: Supplementary file 1 — Supplementary Information. [file 41598_2023_33700_MOESM1_ESM.pdf]

SUPPLEMENTARY INFORMATION

Figure S1. Bubbleplot showing the raw number of individuals dispersing for each sex according to dispersing distance and the number of same-sex elder siblings on : (1) The probability of reproducing; (2) Lifetime fertility for individuals reproducing at least once in their lifetime; (3) Proportion of offspring surviving to age 15. See Methods for details on the study population.

(1) Probability of reproducing

(a) Males

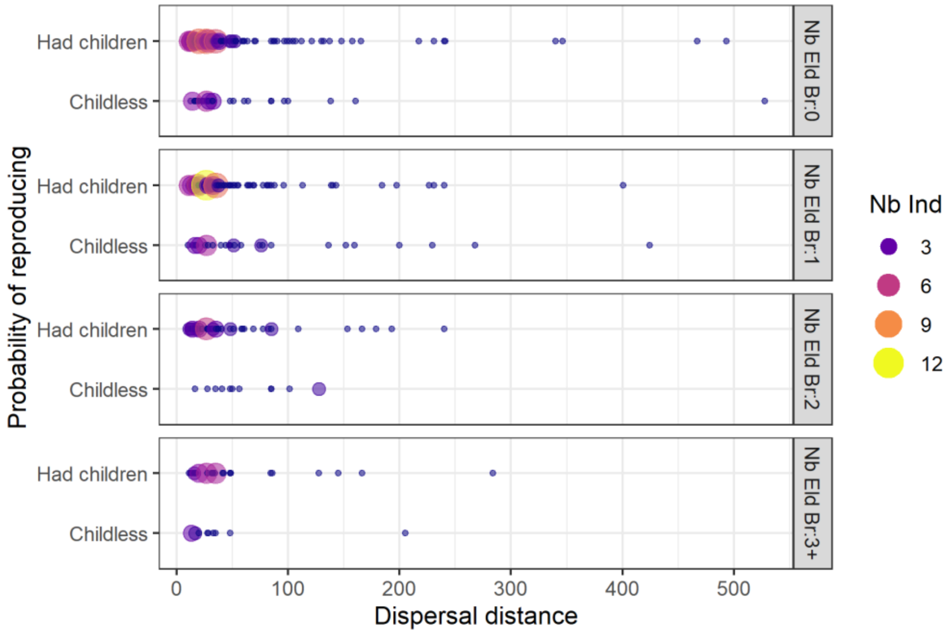

(b) Females

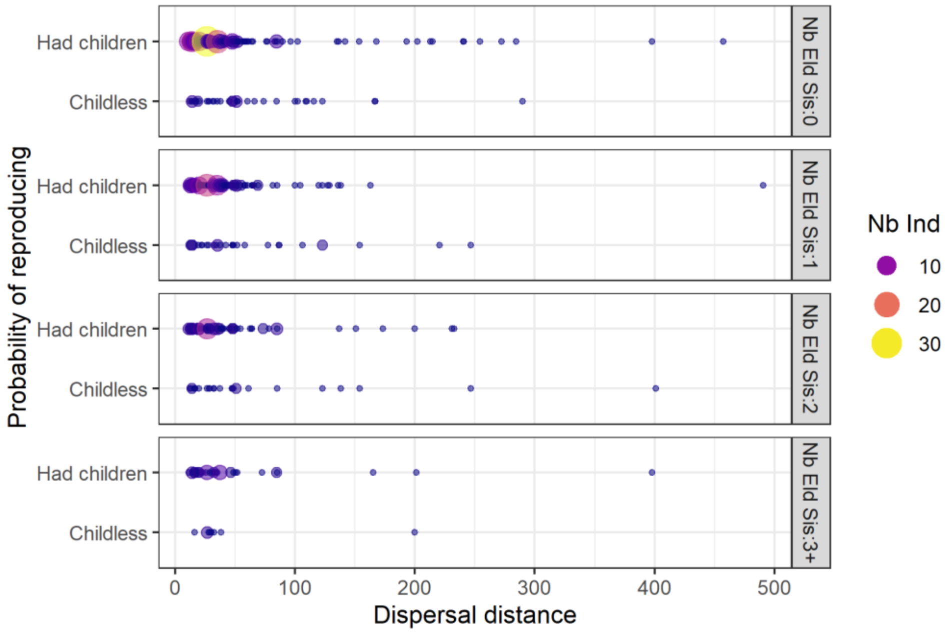

(2) Lifetime fertility

(a) Males

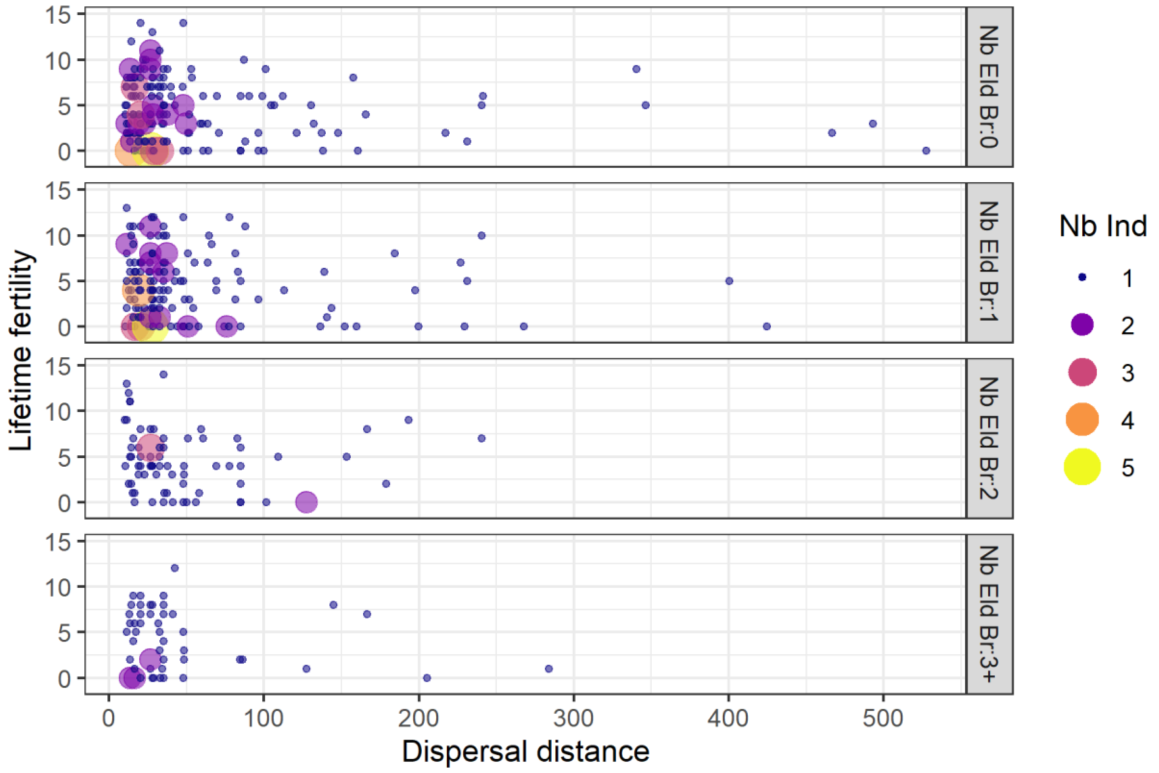

(b) Females

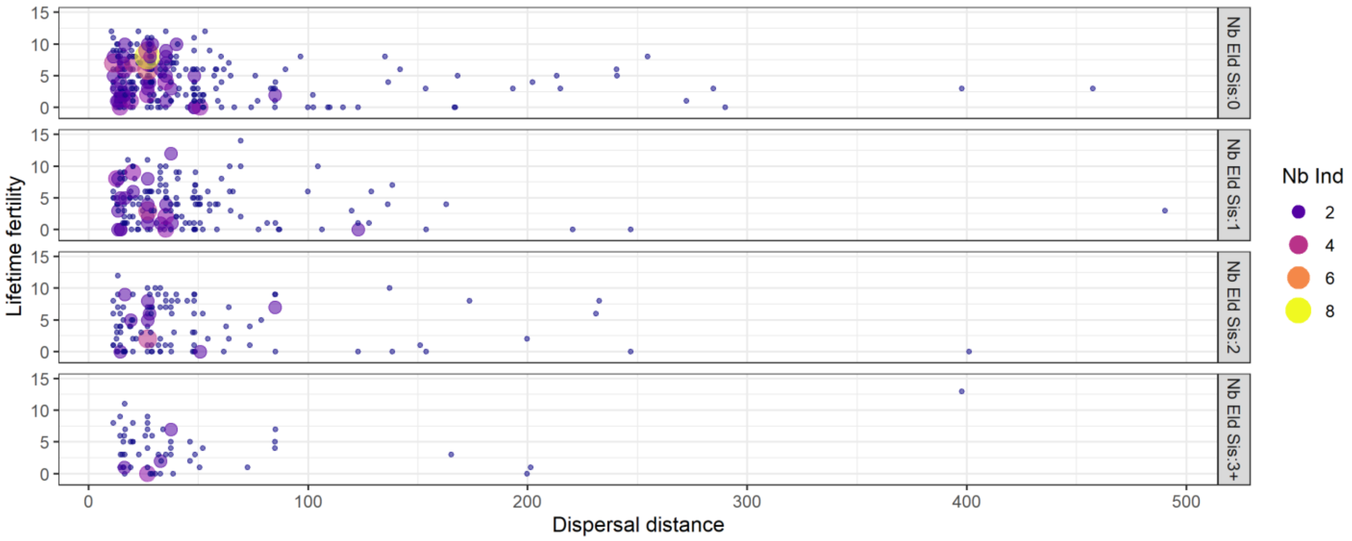

(3) Offpsring survival to age 15

(a) Males

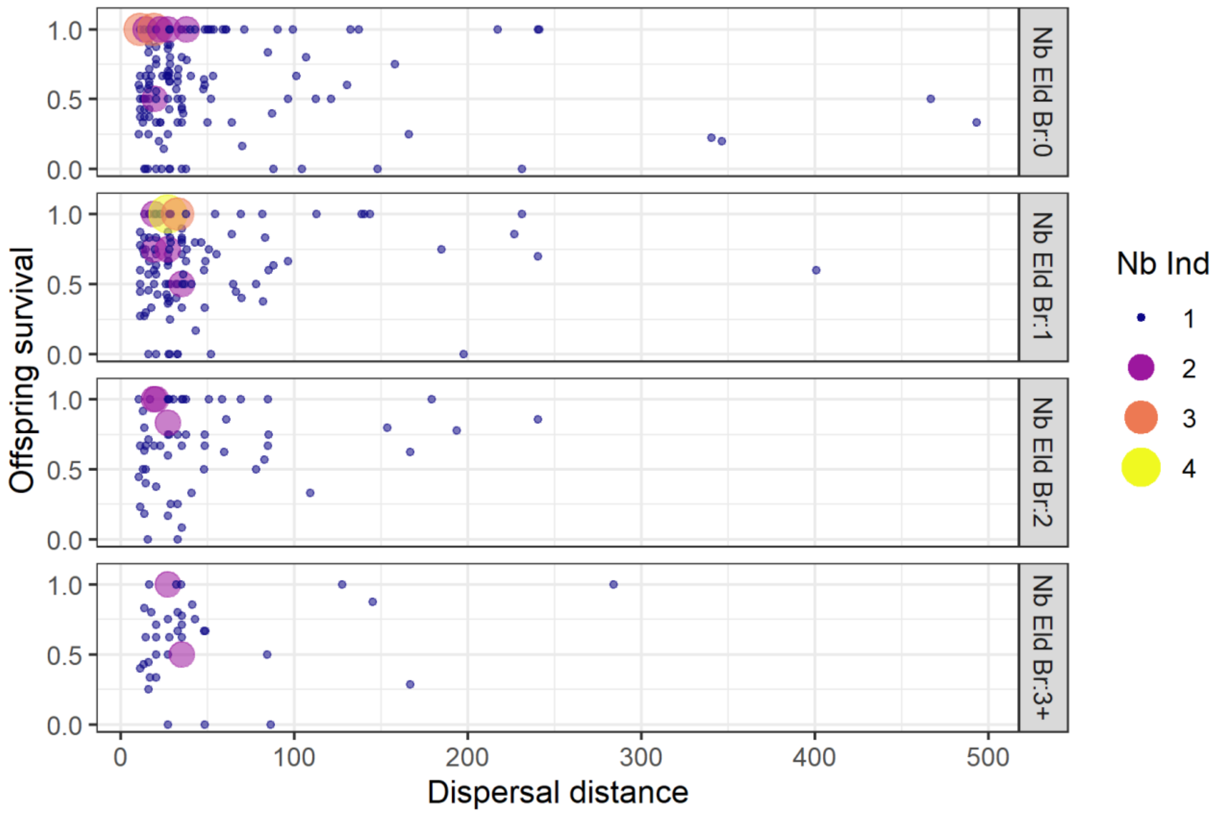

(b) Females

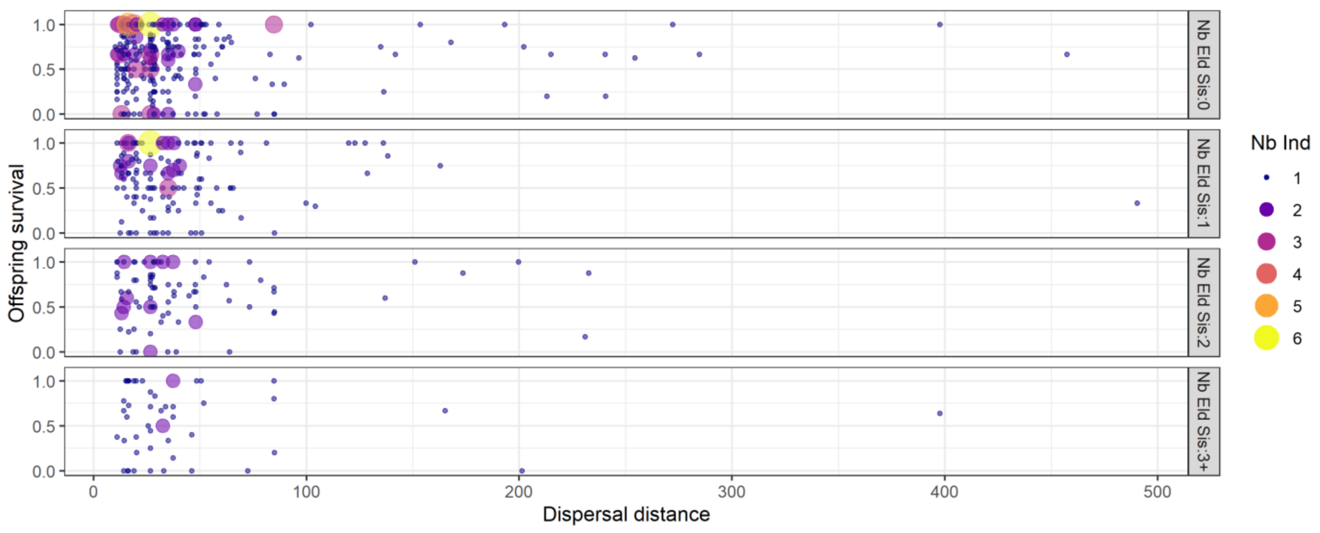

Direct fitness outcomes: Results of the models with dispersal status fitted as a binary variable

Table S1. Probability of reproducing: averaged estimates and their 95% confidence intervals of the best models (full averaging) for (A) males (*N* = 4,485) and (B) females (*N* = 4,529). Models are multi-level logistic regressions, with identity of mother, birth cohort and birth parish included as random effects. "x" denotes an interaction term. "Eld Br" stands for elder brothers, "Eld Sis" for elder sisters and "Disp Status" for lifetime dispersal status. The reference category is underlined for categorical variables. See Methods and Table 2 for details of the model selection results.

(A) Males	Estimate [CI 95%]
Intercept	0.77 [0.56; 0.98]
**Elder brothers**	**-0.25 [-0.36; -0.13]**
Elder sisters	-0.03 [-0.11; 0.04]
Total number of siblings	-0.02 [-0.05; 0.01]
**Low vs. High Fam SES**	**-0.23 [-0.38; -0.09]**
**Dispersed vs. Not Disp**	**0.29 [0.06; 0.51]**
Interaction with Disp Status	
Eld Br x Dispersed	0.10 [-0.13; 0.34]
Interaction with Fam SES	
Eld Br x low SES	0.14 [-0.04; 0.32]

(B) Females	Estimate [CI 95%]
Intercept	1.32 [1.12; 1.51]
Elder brothers	-0.05 [-0.14; 0.03]
**Elder sisters**	**-0.15 [-0.24; -0.05]**
Total number of siblings	-0.01 [-0.05; 0.03]
Low vs. High Fam SES	-0.10 [-0.26; 0.06]
**Dispersed vs. Not Disp**	**0.41 [0.18; 0.63]**
Interaction with Fam SES	
Eld Sis x Low Fam SES	0.01 [-0.08; 0.09]
Interaction with Disp Status	
Eld Sis x Dispersed	-0.03 [-0.18; 0.12]

Table S2. Lifetime fertility: averaged parameter estimates and their 95% confidence intervals from the best models for (A) males (*N* = 3,105) and (B) females (*N* = 3,533). See Methods, Table S1 for details and Table 2 for the model selection results.

(A) Males	Estimate [CI 95%]
Intercept	1.63 [1.56; 1.70]
**Elder brothers**	**-0.07 [-0.10; -0.04]**
Elder sisters	0.00 [-0.02; 0.02]
**Total number of siblings**	**0.02 [0.01; 0.03]**
Low vs. High SES	-0.02 [-0.06; 0.02]
Dispersed vs. Not Disp	-0.02 [-0.08; 0.04]
Interaction with Family SES	
Eld Br x Low SES	0.01 [-0.02; 0.03]
Interaction with Disp Status	
Eld Br x Dispersed	0.03 [-0.04; 0.06]

(B) Females	Estimate [CI 95%]
Intercept	1.59 [1.52; 1.65]
**Elder brothers**	**-0.03 [-0.05; -0.00]**
**Elder sisters**	**-0.04 [-0.06; -0.01]**
Total number of siblings	0.00 [-0.01; 0.01]
**Low vs. High Fam SES**	**-0.07 [-0.11; -0.03]**
Dispersed vs. Not Disp	-0.03 [-0.08; 0.02]
Interaction with Fam SES	
Eld Sis x Low SES	0.02 [-0.02; 0.04]
Interaction with Disp Status	
Eld Sis x Dispersed	0.00 [-0.03; 0.03]

Table S3. Proportion of children born surviving to age 15: odd ratio and 95% confidence intervals from the averaged estimates of the best models for (A) males (*N* = 3,061) and (B) females (*N* = 3,491). See Methods, Table S1 for details and Table 2 for the model selection results.

(A) Males	Estimate [CI 95%]
Intercept	0.94 [0.55; 1.19]
Elder brothers	0.02 [-0.03; 0.04]
Elder sisters	0.01 [-0.03; 0.03]
Total number of siblings	-0.02 [-0.03; 0.01]
Low vs. High SES	-0.05 [-0.09; 0.05]
Dispersed vs. Not Disp	0.00 [-0.07; 0.07]
Interaction with Family SES	
Eld Br x Low SES	0.01 [-0.04; 0.05]

(B) Females	Estimate [CI 95%]
Intercept	0.90 [0.35; 1.32]
Elder brothers	-0.03 [-0.08; 0.01]
**Elder sisters**	**-0.07 [-0.14; 0.00]**
Total number of siblings	0.00 [-0.01; 0.03]
Low vs. High Fam SES	-0.08 [-0.19; 0.03]
Dispersed vs. Not Disp	-0.07 [-0.18; 0.04]
**Interaction with Fam SES**	
**Eld Sis x Low SES**	**0.11 [0.00; 0.20]**
Interaction with Disp Status	
Eld Sis x Dispersed	0.06 [-0.07; 0.16]

Direct fitness outcomes: Results of the models with dispersal status fitted as a 3-level categorical variable

Table S4. Probability of reproducing: averaged estimates and their 95% confidence intervals of the best models (full averaging) for (A) males (N = 4,485) and (B) females (N = 4,529). "Long Dist Disp" stands for long distance dispersal (at least 60 km away from the natal parish) and "Short Dist Disp" for long distance dispersal (less than 60 km away from the natal parish). See Methods, Table S1 for details and Table 3 for the model selection results.

(A) Males	Estimate [CI 95%]
Intercept	0.77 [0.56; 0.98]
**Elder brothers**	**-0.26 [-0.36; -0.14]**
Elder sisters	-0.03 [-0.11; 0.04]
Total number of siblings	-0.02 [-0.05; 0.01]
**Low vs. High Fam SES**	**-0.24 [-0.39; -0.10]**
**Dispersal Cat**	
Long Dist Disp vs. Not Disp	-0.22 [-0.57; 0.13]
**Short Dist Disp vs. Not Disp**	**0.59 [0.31; 0.86]**
Interaction with Fam SES	
Eld Br x Low SES	0.15 [-0.02; 0.33]
Interaction with Disp Cat	
Eld Br x Long Dist Disp	0.06 [-0.16; 0.56]
Eld Br x Short Dist Disp	0.05 [-0.10; 0.44]

(B) Females	Estimate [CI 95%]
Intercept	1.32 [1.13; 1.51]
Elder brothers	-0.05 [-0.13; 0.04]
**Elder sisters**	**-0.15 [-0.24; -0.05]**
Total number of siblings	-0.01 [-0.05; 0.03]
Low vs. High Fam SES	-0.11 [-0.27; 0.05]
**Dispersal Cat**	
Long Dist Disp vs. Not Disp	-0.33 [-0.74; 0.07]
**Short Dist Disp vs. Not Disp**	**0.66 [0.39; 0.92]**
Interaction with Fam SES	
Eld Sis x Low SES	0.01 [-0.07; 0.09]
Interaction with Disp Cat	
Eld Sis x Long Dist Disp	0.01 [-0.17; 0.13]
Eld Sis x Short Dist Disp	-0.03 [-0.16; 0.19]

Table S5. Predicted number of children: averaged parameter estimates and their 95% confidence intervals from the best models for (A) males (*N*= 3,105) and (B) females (*N* = 3,533). See Methods, Table S1 for details and Table 3 for the model selection results.

(A) Males	Estimate [CI 95%]
Intercept	1.63 [1.56; 1.71]
**Elder brothers**	**-0.07 [-0.09; -0.04]**
Elder sisters	0.00 [-0.02; 0.02]
**Total number of siblings**	**0.02 [0.01; 0.03]**
Low vs. High Fam SES	-0.02 [-0.07; 0.02]
**Dispersal status**	
**Long Dist Disp vs. No Disp**	**-0.12 [-0.23; 0.00]**
Short Dist Disp vs. No Disp	0.01 [-0.06; 0.08]
Interaction with Fam SES	
Eld Br x Low SES	0.00 [-0.02; 0.02]
Interaction with Disp Status	
Eld Br x Long Dist Disp	-0.00 [-0.05; 0.05]
Eld Br x Short Dist Disp	0.01 [-0.03; 0.04]

(B) Females	Estimate [CI 95%]
Intercept	1.59 [1.52; 1.65]
**Elder brothers**	**-0.03 [-0.05; -0.004]**
**Elder sisters**	**-0.04 [-0.06; -0.01]**
Total number of siblings	0.00 [-0.01; 0.01]
**Low vs. High Fam SES**	**-0.07 [-0.11; -0.03]**
**Dispersal status**	
**Long Dist Disp vs. Not Disp**	**-0.14 [-0.27; -0.02]**
Short Dist Disp vs. Not Disp	-0.01 [-0.06; 0.05]
Interaction with Fam SES	
Eld Sis x Low SES	0.01 [-0.03 0.04]
Interaction with Disp Status	
Eld Sis x Long Dist Disp	0.02 [-0.07; 0.10]
Eld Sis x Short Dist Disp	-0.00 [-0.03; 0.03]

Table S6. Proportion of children born surviving to age 15: averaged estimates and their 95% confidence intervals of the best models (full averaging) for (A) males (*N* = 3,061) and (B) females (*N* = 3,491). See Methods, Table S1 for details and Table 3 for the model selection results.

(A) Males	Estimate [CI 95%]
Intercept	0.92 [0.78; 1.06]
Elder brothers	0.01 [-0.03; 0.05]
Elder sisters	0.01 [-0.03; 0.04]
Total number of siblings	-0.01 [-0.03; 0.01]
Low vs. High Fam SES	-0.02 [-0.10; 0.06]
Dispersal status	
Long Dist Disp vs. Not Disp	0.09 [-0.17; 0.35]
Short Dist Disp vs. Not Disp	-0.03 [-0.14; 0.08]
Interaction with Fam SES	
Eld Br x Low SES	0.01 [-0.05; 0.06]

(B) Females	Estimate [CI 95%]
Intercept	0.90 [0.76; 1.04]
Elder brothers	-0.04 [-0.08; 0.02]
Elder sisters	-0.08 [-0.13; 0.03]
Total number of siblings	0.01 [-0.01; 0.03]
Low vs. High Fam SES	-0.05 [-0.13; 0.05]
Dispersal status	
Long Dist Disp vs. Not Disp	-0.01 [-0.24; 0.23]
Short Dist Disp vs. Not Disp	-0.10 [-0.20; 0.04]
Interaction with Fam SES	
Eld Sis x Low SES	0.10 [-0.04; 0.19]
Interaction with Disp Status	
Eld Sis x Long Dist Disp	-0.01 [-0.14; 0.12]
Eld Sis x Short Dist Disp	0.08 [-0.07; 0.10]

Indirect fitness outcomes

Table S7. Probability of reproducing: averaged estimates and their 95% confidence intervals of the best models (full averaging) for (A) males (*N* = 1,282) and (B) females (*N* = 1,083). See Methods, Table S1 for details and Table 4 for the model selection results.

(A) Males	Estimate [CI 95%]
Intercept	1.06 [0.68; 1.41]
Low vs. High Fam SES	-0.56 [-0.85; -0.30]
Total number of siblings	0.02 [-0.04; 0.08]

(B) Females	Estimate [CI 95%]
Intercept	1.07 [0.10; 2.04]
Low vs. High Fam SES	-0.28 [-0.64; 0.08]
Total number of siblings	0.04 [-0.03; 0.11]

Table S8. Predicted number of children: averaged parameter estimates and their 95% confidence intervals from the best models for (A) males (*N* = 922) and (B) females (*N* = 835). See Methods, Table S1 for details and Table 3 for the model selection results.

(A) Males		Estimate [95% CI]
Intercept		1.72 [1.62; 1.83]
Low vs. High Fam SES		-0.05 [-0.12; 0.02]
**Total number of siblings**		**0.03 [0.01; 0.05]**
Number of Non-Disp Young Br		-0.00 [-0.03; 0.02]
Proportion of Non-Disp Young Br		
	25-50% vs. 0-25%	0.01 [-0.05; 0.06]
	50-75% vs. 0-25%	0.01 [-0.05; 0.06]
	75-100% vs. 0-25%	-0.00 [-0.06; 0.05]

(B) Females	Estimate [95% CI]
Intercept	1.67 [-0.592; 2.27]
Low vs. High Fam SES	-0.02 [-0.10; 0.05]
Total number of siblings	0.00 [-0.01; 0.02]

Table S9. Proportion of children born surviving to age 15: odd ratio and 95% confidence intervals from the averaged estimates of the best models for (A) males (*N* = 911) and (B) females (*N* = 827). See Methods, Table S1 for details and Table 3 for the model selection results.

(A) Males	Estimate [95% CI]
Intercept	0.16 [-0.50; 0.82]
Low vs. High Fam SES	-0.06 [-0.10; 0.07]
Total number of siblings	0.00 [-0.02; 0.02]

(B) Females	Estimate [95% CI]
Intercept	0.13 [-0.49; 0.75]
Low vs. High Fam SES	-0.01 [-0.08; 0.07]
Total number of siblings	0.00 [-0.02; 0.01]
